# Decreased Bioenergetic Health Index in monocytes isolated from the pericardial fluid and blood of post-operative cardiac surgery patients

**DOI:** 10.1042/BSR20150161

**Published:** 2015-08-07

**Authors:** Philip A. Kramer, Balu K. Chacko, David J. George, Degui Zhi, Chih-Cheng Wei, Louis J. Dell'Italia, Spencer J. Melby, James F. George, Victor M. Darley-Usmar

**Affiliations:** * Mitochondrial Medicine Laboratory, Department of Pathology, University of Alabama at Birmingham, AL 35294, U.S.A.; † Center for Free Radical Biology, Department of Pathology, University of Alabama at Birmingham, AL 35294, U.S.A.; ‡ Department of Biostatistics, University of Alabama at Birmingham, AL 35294, U.S.A.; § Division of Cardiothoracic Surgery, Department of Surgery, University of Alabama at Birmingham, AL 35294, U.S.A.; ║ Department of Veterans Affairs Medical Center, Birmingham, AL 35233, U.S.A.

**Keywords:** Bioenergetic Health Index, cardiac surgery, cardiomyocytes, mitochondria, monocytes, pericardial fluid, oxidative stress

## Abstract

Monitoring the bioenergetics of leucocytes is now emerging as an important approach in translational research to detect mitochondrial dysfunction in blood or other patient samples. Using the mitochondrial stress test, which involves the sequential addition of mitochondrial inhibitors to adherent leucocytes, we have calculated a single value, the Bioenergetic Health Index (BHI), which represents the mitochondrial function in cells isolated from patients. In the present report, we assess the BHI of monocytes isolated from the post-operative blood and post-operative pericardial fluid (PO-PCF) from patients undergoing cardiac surgery. Analysis of the bioenergetics of monocytes isolated from patients’ PO-PCF revealed a profound decrease in mitochondrial function compared with monocytes isolated from their blood or from healthy controls. Further, patient blood monocytes showed no significant difference in the individual energetic parameters from the mitochondrial stress test but, when integrated into the BHI evaluation, there was a significant decrease in BHI compared with healthy control monocytes. These data support the utility of BHI measurements in integrating the individual parameters from the mitochondrial stress test into a single value. Supporting our previous finding that the PO-PCF is pro-oxidant, we found that exposure of rat cardiomyocytes to PO-PCF caused a significant loss of mitochondrial membrane potential and increased reactive oxygen species (ROS). These findings support the hypothesis that integrated measures of bioenergetic health could have prognostic and diagnostic value in translational bioenergetics.

## Abstract (precis)

Translational bioenergetics requires the measurement of mitochondrial function in clinically relevant samples and the integration of the data in a form that can be applied to personalized medicine. In the present study, we show the application of the measurement of the Bioenergetic Health Index (BHI) to cardiac surgery patients.

## INTRODUCTION

Cardiac surgery is a common procedure with a significant incidence of post-operative complications associated with increased morbidity and several complications which have few effective therapies [1–4]. In addition, cardiac surgery inevitably results in varying degrees of cardiac ischaemia, trauma and introduction of blood and other foreign material into the pericardial compartment. We have recently shown that this compartment is a highly pro-oxidant environment with evidence of protein and lipid oxidation [5]. In addition, within a few hours following surgery, the pericardial compartment is populated by monocytes and neutrophils, which we have demonstrated are capable of generating high levels of hydrogen peroxide. Other studies have shown that this pro-inflammatory state may also be reflected to a lesser extent in the systemic circulation. Some inflammatory markers in the serum of post-operative cardiac surgery patients have appeared elevated and correlated with increased occurrences of cardiac dysfunction and atrial fibrillation [6–8]. We have recently proposed that the focus for the initiation of the pro-inflammatory response after surgery is the post-operative pericardial fluid (PO-PCF) and that the oxidative stress in this compartment is more severe than in the blood [5].

Inflammation, increased oxidative stress and ischaemia-reperfusion are known to be associated with mitochondrial dysfunction in the cardiomyocyte and are also thought to be involved in the recruitment of inflammatory cells [9,10]. Recently, we have proposed that inflammatory cells, such as monocytes/macrophages, neutrophils or lymphocytes, can act as a biomarker of inflammation-associated mitochondrial dysfunction defined as changes in cellular bioenergetics [11–13]. Taken together, these findings suggested to us the hypothesis that the monocytes isolated from the PO-PCF, which is more pro-oxidant than the plasma, would exhibit increased mitochondrial dysfunction compared with cells isolated from the patients’ blood. In the present study, we tested this hypothesis in post-operative cardiac surgery patients by isolating monocytes from their PO-PCF and peripheral blood and then comparing their bioenergetic function to the monocytes isolated from the blood of healthy controls. To assess mitochondrial function, we used a mitochondrial stress test in which cells are exposed to a series of mitochondrial inhibitors in sequence, to determine key bioenergetic functions, including mitochondrial reserve capacity, ATP-linked respiration and proton leak [14]. We have shown that the parameters derived from the mitochondrial stress test respond in a co-ordinated fashion under conditions of oxidative stress [15,16]. We have used this approach with the values from the mitochondrial stress test to generate a single value known as the Bioenergetic Health Index (BHI) which provides an index of mitochondrial function in cells [11].

The data indicate that the BHI of monocytes from PO-PCF is strongly depressed relative to monocytes isolated from patient peripheral blood plasma or from the plasma of healthy controls. In addition, more subtle changes in cellular bioenergetics between the blood-derived monocytes from healthy controls and patients were also evident. Taken together, these data further support the concept that the PO-PCF surrounding the heart could promote bioenergetic dysfunction in cardiomyocytes because it is highly pro-oxidant and pro-inflammatory and this, in turn, may contribute to the incidence of post-operative complications, such as atrial fibrillation. As a further test of this concept PO-PCF was incubated with rat cardiomyocytes and found to increase reactive oxygen species (ROS) and decrease mitochondrial membrane potential. The data also support the hypothesis that BHI values in cells from the PO-PCF could potentially serve as a marker or predictor of events occurring in or proximal to the heart.

## MATERIALS AND METHODS

### Surgery, blood collection and cell isolation

All study protocols for collection and handling of human samples were reviewed and approved by the Institutional Review Board, University of Alabama at Birmingham. Consent was obtained from healthy adult donors (10 male, three female, 26–62 years of age) or adult patients [eight male, six female (38–83 years of age)], undergoing cardiac surgery for ischaemic heart disease or valvular heart disease: coronary artery bypass graft ± aortic valve replacement/mitral valve repair/replacement, tricuspid valve repair or replacement or a valve procedure alone. Patients with ventricular assist devices, atrial fibrillation surgery, thoracic aorta surgery, concomitant non-cardiac surgery and patients with atrial fibrillation within 6 months were excluded.

Blood samples (1–2 tubes, 8.5 ml/tube) were collected from 13 healthy donors and 14 cardiac surgery patients 4–12 h after surgery. Blood was collected in vacutainers (BD Biosciences) containing 1.5 ml of ACD (acid citrate dextrose) solution (trisodium citrate, 22.0 g/l; citric acid, 8.0 g/l and dextrose 24.5 g/l) as anti-coagulant and processed within 2 h of collection using an established protocol [17]. All isolation procedures were designed to prevent activation of the cells during isolation and were performed at room temperature. In a paired study design PO-PCF was obtained and assayed in parallel with peripheral blood drawn from each patient at the same time point. PO-PCF was allowed to accumulate no more than 1 h prior to collection in the mediastinal drainage tube. After collection, the blood and PO-PCF were centrifuged at 500 g for 10 min at room temperature. The leucocyte-enriched layer on top of the RBC (red blood cell) pellet (buffy coat), was diluted (1:4) with basal RPMI (Roswell Park Memorial Institute) media and applied to a Histopaque density gradient (specific gravity=1.077/1.119, at room temperature, Sigma Chemical Co.) and centrifuged without application of the brake at 700 ***g*** for 30 min. The peripheral blood mononuclear cells (PBMCs) and polymorphonuclear cells (granulocytes, PMN) were collected separately. CD14^+^ monocytes were purified from PBMC fraction by the MACS (magnetic-activated cell sorting) technique (Milteneyi Biotec) using superparamagnetic iron-dextran microbead-labelled anti-CD14 antibodies according to manufacturer's instructions. Briefly, PBMC were incubated with the labelled anti-CD14 antibodies at 4°C–8°C for 15 min prior to applying the cells to the column placed in the magnetic field. Cells retained in the column were collected by eluting with RPMI containing 0.5% BSA after removing from the magnetic field and used for analysis as described below. Isolation yielded cell populations with >90% viability as determined by Trypan Blue exclusion (result not shown).

### Bioenergetic assessment of CD14+ monocytes

Determination of cellular bioenergetics was performed after plating the cells on 24-well polystyrene plates designed for the extracellular flux (XF) analyser [14]. Purified monocytes were resuspended in XF assay buffer and plated the diluted cells (250000 cells/well) in 200 μl on CellTak (BD Biosciences) coated assay plates and allowed to attach for 30 min at 37°C in a non-CO_2_ incubator. The cellular bioenergetics of the isolated cells was determined using the XF analyser (Seahorse Bioscience) in combination with the mitochondrial stress test [17]. Real-time, non-invasive measurements of oxygen consumption rate (OCR) and extracellular acidification rate (ECAR) were measured and correlated to mitochondrial function and glycolysis respectively. The percent of non-mitochondrial and glycolytic monocyte OCR and ECAR were assessed in the XF96 XF analyser (Seahorse Bioscience) as a glucose stress test with sequential injections of glucose (5 mM), oligomycin (1.0 μg/ml) and 2-deoxyglucose (100 mM) (Supplementary Figure S1). Using the mitochondrial stress test protocol, inhibitors of the mitochondrial respiratory chain were injected sequentially to assess the respiratory parameters: basal OCR, ATP-linked OCR, proton leak, maximal and non-mitochondrial OCR. Reserve capacity was calculated by the subtraction of basal OCR from maximal OCR. The optimum concentration of the inhibitors and activators to be used for the assessment of mitochondrial function were determined, as previously described [14,18]. All XF assays were performed in sterile DMEM (Dulbecco's modified Eagle's medium; pH 7.4) containing 5 mM D-glucose, 4 mM L-glutamine and 1 mM sodium pyruvate. The BHI was calculated using the following equation: (Reserve capacity × ATP-linked OCR)/(Proton leak × Non-mitochondrial OCR). No significant correlation was found between age and gender for BHI between either the patient or the healthy control groups. For samples in which there was no significant antimycin-inhibitable OCR or a minimal antimycin and no significant oligomycin OCR, the BHI was considered zero. BHI values were also excluded in individual samples that had a minimal to undetectable proton leak or non-mitochondrial OCR due to the limits of detection of the instrument. Using these criteria, two BHI values were completely excluded from the patient blood and PO-PCF groups. Under the conditions of the mitochondrial stress test the viability was over 90% and remained so over the time course of the assay. At the end of the assay period, lysis buffer was added to each well and assayed for protein by the DC Lowry (Bio-Rad) protein assay. OCR and ECAR values were normalized to the protein content in each well.

Mitochondrial complex I and II activities were assessed by cell membrane permeabilization as previously described with an equal number of monocytes for each condition [19]. Since protein is lost from the cells, on permeabilization the date is not normalized and was not found to be significantly different on a parallel plate used for the mitochondrial stress test. Monocytes isolation and plating was performed as described above, however cells were incubated in mannitol and sucrose (MAS) buffer (70 mM sucrose, 220 mM mannitol, 10 mM KH_2_PO_4_, 5 mM MgCl_2_, 2 mM HEPES, 1 mM EGTA, pH 7.2) instead of XF–DMEM for the assay. After three basal OCR measurements were acquired, saponin (30 μg/ml), pyruvate (5 mM), malate (2.5 mM), succinate (10 mM) and ADP (1 mM) were injected. A maximal OCR measurement was obtained followed by injection of rotenone (1 μM) and subsequently antimycin A (10 μM) to determine complex I and complex II linked respiration.

### Isolation of rat cardiomyocytes and exposure to PO-PCF

Primary adult rat cardiac myocytes were isolated from untreated rats (250–300 g) using the modified protocol as described previously [20]. This part of the study was approved by the University of Alabama at Birmingham Institutional Animal Care and Use Committee. Briefly, the hearts were harvested from isoflurane anaesthetized experimental animals and perfused with the perfusion buffer (120 mM NaCl, 15 mM KCl, 0.5 mM KH_2_PO_4_, 5 M NaHCO_3_, 10 mM HEPES and 5 mM glucose, pH 7.0) for 5 min to remove blood and the plasma components. The perfused hearts were digested with perfusion buffer containing 2% collagenase II (Life Technologies) for 30 min at 37°C and the left ventricles were carefully collected by removing the right ventricle, the atria and the apex and minced. The single cell suspension was washed with gentle centrifugation and the pellets were collected. The cardiomyocytes preparations that yielded very high purity (>95%) and a viability of more than 80% (rod-shaped) only were used for the studies using PO-PCF. The cardiomyocytes were attached on laminin (5 μg/ml)-coated glass-bottom chamber slides (10000/cm^2^) were exposed to either 5% cell-free PO-PCF (4 h post-surgery) or plasma for 3 h at 37°C in a 5% CO_2_ incubator.

### Determination of ROS and mitochondrial membrane potential in cardiomyocytes

Prior to the determination of ROS/mitochondrial membrane potential, the cells were gently washed to remove the treatment medium and loaded with DCF (2′,7′-dichlorodihydrofluorescein diacetate 1.0 μM, Life Technologies) or JC-1 (0.5 μM, Life Technologies) in basal DMEM medium (without serum) for 15 min at 37°C. The control plasma samples were diluted appropriately to the protein concentration equivalent to that of the corresponding PO-PCF treatment. After removing the fluorescent probe-containing media, the cells were imaged using a fluorescent microscope (Leica DMRB) and the fluorescent intensities were determined using Simple PCI software (Vashaw Scientific). Red to green fluorescence intensities of individual PO-PCF treated cardiomyocytes were determined and compared with the plasma treated cells.

### Statistics

Each sample was analysed with 3–5 replicates for mitochondrial bioenergetics and data are presented as mean ± S.E.M. Statistical significance was determined using an unpaired Student's *t* test, for the comparison between healthy controls and cardiac surgery blood monocytes and a paired *t* test for the comparison between blood-derived and PO-PCF monocytes from the cardiac surgery patients. *P*<0.05 was taken as significantly different. A *post-hoc* power analysis was performed for the BHI analysis and based on the effect sizes, power was estimated to be a minimum of 99.6% for all group comparisons.

## RESULTS

### Monocyte bioenergetic function in peripheral blood and pericardial fluid

PO-PCF and blood was collected at the same time and within 12 h after cardiac surgery. CD14+ monocytes isolated from these patient samples were then subjected to the mitochondrial stress test which was performed within 1 h after plating. Injections of oligomycin (0.5 μg/ml), FCCP (carbonyl cyanide 4-(trifluoromethoxy)phenylhydrazone 1 μM) and antimycin A (10 μM), were used to obtain the mitochondrial function profiles as previously described [17]. Figure 1(A) depicts representative bioenergetic profiles of patient monocytes isolated from the blood and PO-PCF as well as the healthy donor monocyte mitochondrial profile. In the healthy control monocytes, the expected decrease in basal OCR occurs on addition of oligomycin followed by the stimulation of OCR by FCCP and its inhibition by antimycin A. The monocytes isolated from patients’ blood show small differences in the profile, which, when averaged over all the subjects, are not significantly different (Figure 1B). In contrast, a significant suppression of all mitochondrial parameters is observed in the mitochondria of the monocytes isolated from post-operative PCF (Figures 1A and 1B). Specifically, basal, ATP-linked, proton leak, reserve capacity and maximal mitochondrial OCR were significantly decreased in PO-PCF monocytes relative to their matched blood and healthy donor blood monocytes. No significant differences were observed in non-mitochondrial OCR.

**Figure 1:**
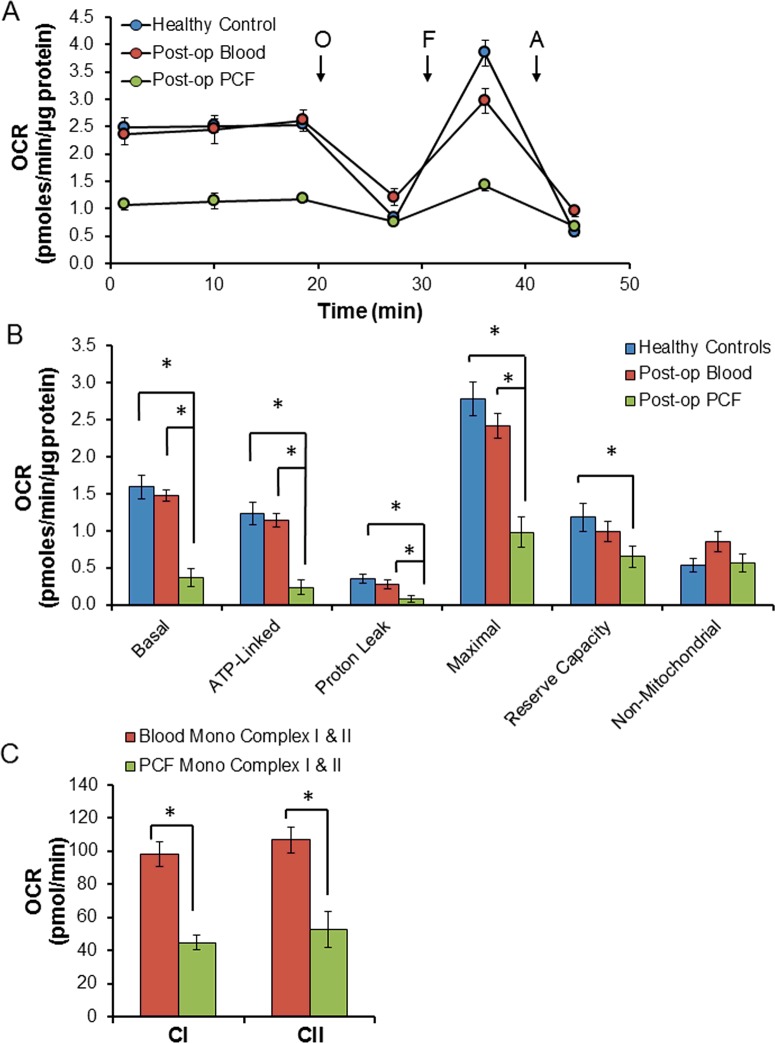
Monocyte bioenergetics health in post-operative patient PO-PCF and blood Monocytes were isolated by positive selection using anti-CD14 miltenyi microbeads and MACS isolation from matched pericardial fluid and blood collected within 12 h following surgery and plated on the Seahorse XF24 at 250000 cells/well. (**A**) The representative patient and healthy donor profiles showing OCR over 45 min with the addition of oligomycin (O), FCCP (F) and antimycin A. (**B**) The average bioenergetic indices were calculated in 13 patients and 13 healthy controls and compared for significance. (**C**) Monocytes from a single patient's blood and PO-PCF were permeabilized (30 μg/ml saponin) and mitochondria exposed to excess complex I and complex II linked substrates. Complex I-linked OCR (CI) and complex II-linked OCR (CII) were obtained by injection of rotenone (1 μM) and antimycin A (10 μM), respectively; *P<0.05. Data represent mean ± S.E.M. *n*=3–5 replicate wells per sample using an unpaired Student's *t* test to compare healthy controls to the patient groups and a paired Student's *t* test to analyse significance between matched patient samples.

To determine if the mitochondrial dysfunction in PO-PCF monocytes was due to decreased substrate uptake, utilization or damage to the respiratory chain, a mitochondrial permeabilization assay was performed. Selective permeabilization of the plasma membrane was achieved by the addition of saponin (30 μg/ml) in MAS buffer after which exogenous respiratory substrates (ADP, succinate, pyruvate and malate) were delivered to the mitochondria. Basal OCR was measured prior to cell permeabilization, followed by measurement of maximal mitochondrial function on addition of the substrates and then rotenone (1 μM) and antimycin A (10 μM) were injected to inhibit electron transport chain complex I and complex II activity respectively. Figure 1(C) shows the complex I and II linked respiration in monocytes isolated from the blood and PO-PCF of a single patient. Mitochondria in blood monocytes utilized approximately 50% complex I and 50% complex II (as determined by antimycin A) in non-substrate limiting conditions and were similar in monocytes isolated from PO-PCF. However, the maximal OCR linked to complex I and complex II was significantly lower in PO-PCF isolated monocytes.

### Measurement of the Bioenergetic Health Index

We have recently demonstrated that the individual parameters from the mitochondrial stress test are interactive and that they can be integrated into a single value representing the profiles shown in Figures 1(A) and 1(B) known as the BHI [11]. As shown in Figure 2(A), BHI is significantly different (*P*=0.030) between the healthy controls and the monocytes isolated from the blood from patients following surgery. Although each bioenergetics parameter measured (Figures 1A and 1B) was not significantly different between blood analysis of healthy donors and blood from cardiac surgery patients when compared independently, when integration of bioenergetic parameters using the BHI calculation is performed, a significant difference is now apparent (Figure 2A) [11]. This arises from the fact that the energetics parameters in the mitochondrial stress test are interactive and small changes in individual parameters cannot capture the overall change in the bioenergetic profile in response to stress. In support of our hypothesis that the post-operative pericardial environment can damage mitochondrial function, the monocytes isolated from the PO-PCF show the lowest value for BHI, which is also significantly different from both the monocytes isolated from the patients’ blood (*P*=0.004) and the healthy controls (*P*=0.001). As an additional test of the impact of the PO-PCF, the paired BHI data for each individual cardiac surgery patient is shown in Figure 2(B). Of the nine patients shown, seven showed a highly significant decrease in BHI in the monocytes isolated from PO-PCF compared with controls. Because there is variation in the BHI in the monocytes from the patient's blood these data are re-plotted in Figure 2(C) where the BHI for the patients’ monocytes has been set at 100% and the percentage change in BHI for the monocytes isolated from the PO-PCF calculated. For all but two of the patients, the BHI value for the PO-PCF monocytes compared with those isolated from the blood is decreased more than 70%. Interestingly, the patients with some of the lowest BHI values in peripheral blood were also those that developed atrial fibrillation, a common post-operative cardiac arrhythmia (Figure 2D).

**Figure 2:**
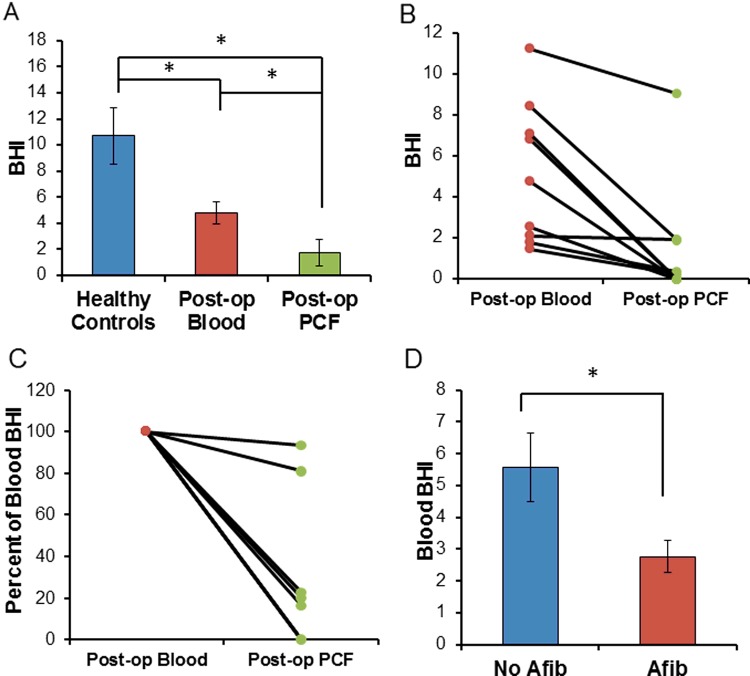
BHI in cardiac surgery patients (**A**) The BHI was calculated using the equation: mitochondrial (Reserve Capacity × ATP-linked OCR)/(Proton Leak × Non-mitochondrial OCR) for 13 healthy controls and 11 patient matched blood monocytes and the monocytes isolated from the PO-PCF. (**B**) BHI in individual patients’ blood and PO-PCF. (**C**) The BHI for the patient's blood monocytes was established as 100% and used to calculate the percentage BHI in the same patients PO-PCF. (**D**) The association of blood BHI in monocytes was significant in patients that developed atrial fibrillation (*n*=3) compared with the BHI of those that did not (*n*=8). *P<0.05. Data represent mean ± S.E.M. *n*=3–5 replicate wells per sample using an unpaired Student's *t* test for the comparison of healthy controls with patient monocytes from PO-PCF and blood and a paired Student's *t* test for comparison between the same patient's blood and PO-PCF monocytes.

### Mitochondrial and glycolytic dysfunction in monocytes isolated from PO-PCF

The changes in ECAR which were measured in parallel to the mitochondrial stress test are shown in Figure 3(A) for a representative patient and normal subject. On the basis of the glycolysis stress test, we found that approximately 72% of the observed basal ECAR can be attributed to glycolysis (Supplementary Figure S1). Monocytes can modulate their metabolic function on activation and become less oxidative and more glycolytic [12]. This can be visualized by plotting the basal OCR against the basal ECAR for each monocyte group using the data obtained in Figure 1. As shown in Figure 3(B) and as we have demonstrated previously, blood monocytes exhibit an aerobic glycolysis phenotype which is not significantly different in the monocytes isolated from the patient's blood [13]. In contrast, the monocytes isolated from the PO-PCF show significantly depressed basal OCR without a significant change in basal ECAR when compared with monocytes from health controls or the same patients. The addition of oligomycin blocks mitochondrial ATP synthase and stimulates ECAR, which in monocytes we have shown can be ascribed to glycolysis [13]. Figure 3(C) shows the oligomycin-sensitive changes in OCR and ECAR which directly reflect ATP-linked respiration and the capacity to induce glycolysis. These data show that oligomycin fails to stimulate glycolysis in the monocytes isolated from the PO-PCF and that ATP-linked OCR is significantly depressed compared with healthy control or patient blood monocytes.

**Figure 3:**
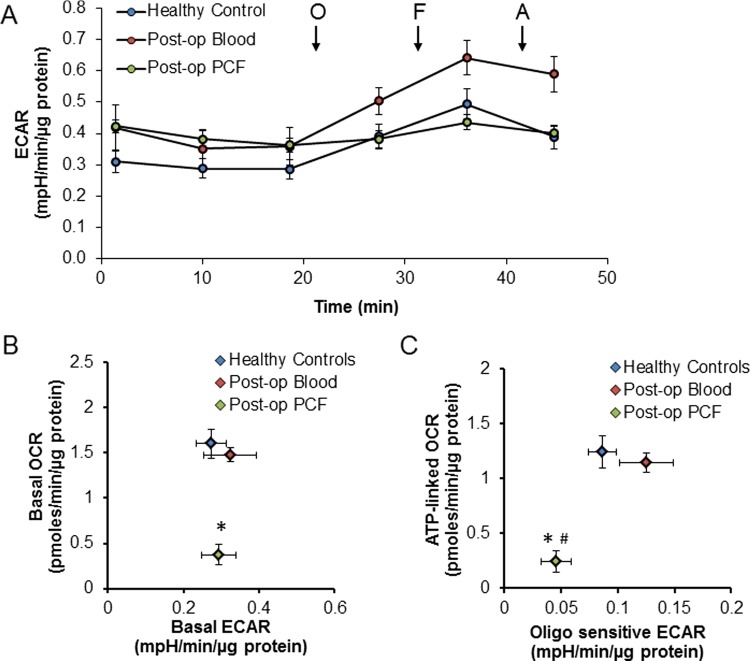
Oxidative phosphorylation and glycolytic dysfunction in pericardial fluid monocytes (**A**) Representative patient ECAR traces are shown in each patient group for the mitochondrial stress test, consisting of oligomycin (0.5 μg/ml), FCCP (0.6 μM) and antimycin A (1.0 μg/ml). (**B**) Monocyte OCR and ECAR represent the basal oxidative phosphorylation and glycolysis in blood and pericardial fluid of patients and the blood of healthy controls. (**C**) Following oligomycin (0.5 μg/ml), OCR/ECAR plots show the ATP-linked oxygen consumption and glycolysis. Data represent mean ± S.E.M. *n*=3–5 replicate wells per sample. *n*=13 healthy controls and 13 post-operative patients. * and # denotes a significant change from control in OCR and ECAR respectively (*P*≤0.05). Significance was measured using an unpaired Student's *t* test to compare healthy controls to the patient groups and a paired Student's *t* test to analyse significance between matched patient samples.

### PO-PCF induces oxidative stress and mitochondrial damage in cardiomyocytes

We have previously shown that PO-PCF is a highly pro-oxidant environment within the pericardial space surrounding the heart [5]. In addition, the PO-PCF contains high levels of cardiomyocyte proteins indicating that the epicardial barrier has been breached allowing contact between the PO-PCF and the cardiomyocytes. To address the impact of PO-PCF on adult rat cardiomyocytes, cells in XF–DMEM were treated with 5% PO-PCF or 5% plasma from the same patient. Both were collected 4 h after surgery. PO-PCF caused a dramatic increase in oxidative stress in cardiomyocytes as determined by DCF fluorescence assay (Figure 4A) compared with the plasma control from the same patient. Mitochondria are one of the most sensitive targets of ROS and several studies demonstrate mitochondrial dysfunction via oxidation of mitochondrial proteins, inhibition of membrane potential and damage to the organelle [14,21,22]. *In vitro* PO-PCF treatment of cardiomyocytes also caused a decrease in mitochondrial membrane potential as determined by the poor dimerisation of JC-1 in the mitochondria (Figures 4B and 4D).

**Figure 4:**
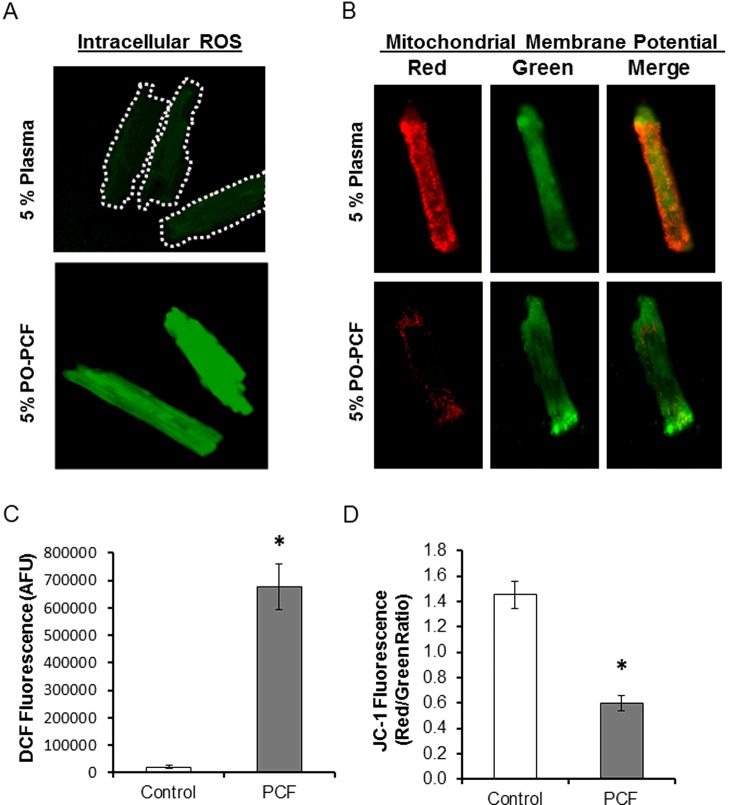
PO-PCF induces oxidative stress in cardiomyocytes and suppresses mitochondrial membrane potential Exposure of isolated adult rat ventricular cardiomyocytes to cell-free PO-PCF (5%) or plasma from the same patient (3 h, 37°C, 5% CO_2_) induced oxidative stress in cardiomyocytes as determined using DCF-DA fluorescence (**A**) and expressed as fluorescence per cell (**C**). Mitochondrial membrane potential in cardiomyocytes exposed to PO-PCF was determined using JC-1 assay (0.5 μM) (**B**) and the red/green fluorescence ratio was assessed per cell (**D**). All the images were captured using a Leica fluorescence microscope (Total magnification 100×). Mean values from 4–8 replicates were plotted ± S.E.M. *P≤0.001 by Student's *t* test.

## DISCUSSION

Cardiac surgery has both systemic and local effects and the trauma of the surgery profoundly alters the composition of the PCF surrounding the heart. The changes in the composition and volume of the pericardial fluid that occur post-surgery, include the accumulation of inflammatory cells, pro-oxidants including hydrogen peroxide and oxidized lipids which could alter cardiac mitochondrial function [5,10]. An important concept in cellular bioenergetics is that leucocytes and platelets can act as biomarkers for mitochondrial dysfunction where tissue collection is not feasible [23]. In the present study, the primary hypothesis we addressed is whether the monocytes exposed to the pro-oxidant environment of the PO-PCF induces bioenergetic dysfunction compared with monocytes isolated from the same patient's blood.

Oxidized haemoglobin (Hb) and lipid peroxidation products are found at high levels of in the PO-PCF but to a much lower extent in the blood [5]. Both oxidized lipids and hydrogen peroxide are known to damage mitochondria [14,22]. For example, mitochondrial dysfunction is a major consequence of lipid peroxidation, promoted by haeme and haeme proteins and is characterized by irreversible permeabilization of the mitochondrial membrane, a decrease in membrane potential, loss of cellular bioenergetics and mitochondrial swelling [24–28]. These findings suggest that the severity of any mitochondrial defects correspond to the greatest exposure to oxidative stress, which in this case would be in the PO-PCF. The monocytes isolated from the PO-PCF exhibited lower complex I and II linked respiration (Figure 1C) consistent with inhibition at complexes III, IV or V. Further, the exposure of rat cardiomyocytes to PO-PCF increased ROS and decreased mitochondrial membrane potential significantly more than blood plasma (Figure 4). These data are consistent with the capability of the PO-PCF to induce bioenergetic dysfunction. The factors mediating these effects are unknown and will require more investigation.

We found that the monocytes isolated from the PO-PCF showed a decrease in all bioenergetic parameters (Figure 1) and also displayed suppressed glycolytic activity (Figure 3). Interestingly, monocytes and macrophages exhibit a metabolic switch associated with the phenotypic switch to the pro-inflammatory M1 phenotype which is characterized by suppressed mitochondrial function [12,29]. To what extent these changes contribute to the modified bioenergetics in the monocytes isolated from PO-PCF is unclear and will require further investigation. The changes in cellular bioenergetics in the monocytes from healthy controls when compared with monocytes from the blood of the patients are more subtle and overall show no significant difference in any individual bioenergetic parameter (Figure 1). We have recently demonstrated that the bioenergetic parameters in the mitochondrial stress test are interactive and synergistic. This has led to the proposal that an integrated assessment of mitochondrial function would be a more sensitive and accurate indicator of a patient's bioenergetic health [11]. To test this, we determined the BHI from the data shown in Figure 1(A) and found that, in contrast with the individual parameters from the mitochondrial stress test that BHI was decreased in the patients’ monocytes relative to healthy controls (Figure 2). Interestingly, it showed the lowest values in the cells isolated from the PO-PCF compared with BHI in blood monocytes from the same patient and was suppressed over 70% in the PO-PCF.

Limitations of the present study include the possibility that medications in the surgery patients may affect bioenergetics and this could contribute to the differences between control and healthy subjects. The control subjects were not age or gender matched in the present pilot study but we have found in more extensive studies we have found no relationship of either age or gender with bioenergetic parameters. However, this is unlikely to be a major factor in the comparison between the monocytes isolated from the PO-PCF when compared with the patient's own blood. In addition, the probes for the measurement of ROS also have their limitations including the possibility that increased iron contributed to the increased DCF signal in the cardiomyocytes incubated with PO-PCF [30].

It is important to recognize that this may not be the optimal BHI equation to identify those patients most at risk for post-operative complications. However, it is notable that the three patients who exhibited this complication had among the lowest BHI values. These data also support the utility of the BHI calculation as a biomarker of bioenergetic dysfunction in patient populations. These data support our hypothesis that, following cardiac surgery, the area immediately surrounding the heart is the most pro-oxidant environment in the patient and can potentially damage mitochondrial function. In summary, these preliminary data encourage longitudinal studies to determine if there is a significant association between the oxidative stress, inflammation and bioenergetic dysfunction and atrial fibrillation and other post-operative complications, using blood and PCF monocytes as a surrogate marker of mitochondrial and glycolytic dysfunction.

## Abbreviations

(BHI): Bioenergetic health index
DMEM: dulbecco's modified eagle's medium
(ECAR): extracellular acidification rate
MAS: mannitol and sucrose
OCR: oxygen consumption rate
(PBMC): peripheral blood mononuclear cell
(PO-PCF): post-operative pericardial fluid
(ROS): reactive oxygen species
RPMI: Roswell Park Memorial Institute
(OCR): oxygen consumption rate
